# Scrapie infectivity is quickly cleared in tissues of orally-infected farmed fish

**DOI:** 10.1186/1746-6148-2-21

**Published:** 2006-06-15

**Authors:** Loredana Ingrosso, Beatriz Novoa, Andrea Z Dalla Valle, Franco Cardone, Raquel Aranguren, Marco Sbriccoli, Simona Bevivino, Marcello Iriti, Quanguo Liu, Vito Vetrugno, Mei Lu, Franco Faoro, Salvatore Ciappellano, Antonio Figueras, Maurizio Pocchiari

**Affiliations:** 1 Istituto Superiore di Sanità, Department of Cellular Biology and Neuroscience, viale Regina Elena,299,00161 Rome, Italy; 2 Instituto Investigaciones Marinas, CSIC, Eduardo Cabello 6, 36208 Vigo, Spain; 3 Section of Human Nutrition, Department of Food Science and Microbiology, DiSTAM, University of Milan, via Celoria 2, 20133 Milano, Italy; 4 Institute of Plant Pathology, University of Milan and Institute of Plant Virology, CNR, Milano, Italy

## Abstract

**Background:**

Scrapie and bovine spongiform encephalopathy (BSE) belongs to the group of animal transmissible spongiform encephalopathy (TSE). BSE epidemic in the UK and elsewhere in Europe has been linked to the use of bovine meat and bone meals (MBM) in the feeding of cattle. There is concern that pigs, poultry and fish bred for human consumption and fed with infected MBM would eventually develop BSE or carry residual infectivity without disease. Although there has been no evidence of infection in these species, experimental data on the susceptibility to the BSE agent of farm animals other than sheep and cow are limited only to pigs and domestic chicken. In the framework of a EU-granted project we have challenged two species of fish largely used in human food consumption, rainbow trout (*Oncorhynchus mykiss*) and turbot (*Scophthalmus maximus*), with a mouse-adapted TSE strain (scrapie 139A), to assess the risk related to oral consumption of TSE contaminated food. In trout, we also checked the "in vitro" ability of the pathological isoform of the mouse prion protein (PrP^Sc^) to cross the intestinal epithelium when added to the mucosal side of everted intestine.

**Results:**

Fish challenged with a large amount of scrapie mouse brain homogenate by either oral or parenteral routes, showed the ability to clear the majority of infectivity load. None of the fish tissues taken at different time points after oral or parenteral inoculation was able to provoke scrapie disease after intracerebral inoculation in recipient mice. However, a few recipient mice were positive for PrP^Sc ^and spongiform lesions in the brain. We also showed a specific binding of PrP^Sc ^to the mucosal side of fish intestine in the absence of an active uptake of the prion protein through the intestinal wall.

**Conclusion:**

These results indicate that scrapie 139A, and possibly BSE, is quickly removed from fish tissues despite evidence of a prion like protein in fish and of a specific binding of PrP^Sc ^to the mucosal side of fish intestine.

## Background

Transmissible spongiform encephalopathy (TSE) or prion diseases are fatal human and animal neurological disorders with a worldwide distribution. Human TSE diseases include sporadic, genetic, iatrogenic and variant Creutzfeldt-Jakob disease (CJD), Gerstmann-Sträussler-Scheinker disease and sporadic or familial fatal insomnia. Animal counterparts are scrapie in sheep and goats, bovine spongiform encephalopathy (BSE), transmissible mink encephalopathy, and chronic wasting disease of mule deer and elk. There are strong evidences that among human TSE diseases only variant CJD is caused by the consumption of BSE-contaminated meat products [1,2]. The critical pathogenetic event in TSE diseases is the conformational change of the physiological host prion protein (PrP^c^) into an insoluble form (PrP^Sc^) able to provoke the pathognomonic brain lesions and death. Transgenic mice devoid of PrP^c ^are unable to sustain TSE infection after experimental inoculation demonstrating the key role of PrP^c ^in the pathogenesis of these diseases [3].

The PrP gene is highly preserved among mammals [4], and sequences of prion-like cDNAs have been described in other vertebrate classes including birds [5-7], reptiles [8,9], amphibians [10], and fish [11-13]. The presence of proteins "similar to" PrP^c ^(stPrP, [12]) in fish has raised concern about a possible transmission of TSE agents to humans through consumption of farmed fish since mammalian MBM (meat and bone meal) and other mammalian products were historically fed to farmed fish [14]. The distribution of stPrP in trout organism was also studied through the use of newly described monoclonal antibodies which show that the protein is preferentially distributed in brain, optic nerve and spinal cord in contrast to its absence (or presence at undetectable level) outside the nervous system, including the intestine [15].

The passage of TSE agents between animals of different species is usually impaired by what is called the species barrier, i.e. the difficulty to establish clinical disease into the new host even after a prolonged incubation period. Infectivity, however, might be present without clinical presentation of disease, and tissues from first attempted transmission might be infectious when re-inoculated in susceptible animals [16].

The need to give an answer to public concern about safety of food possibly contaminated with TSE agents prompted us to set up an experiment that uses fish as recipient of a scrapie agent (mouse-adapted 139A strain). Both "in vitro" and "in vivo" approaches were devised in an attempt to draw a pattern of risk related to human consumption of fish products. The 139A mouse-adapted TSE scrapie strain was chosen because of its ability to cross the species barrier in different species of rodents [17], while trout (*Oncorhynchus mykiss*) and turbot (*Scophthalmus maximus*) for their large use in aquaculture food industry. We also challenged the "in vitro" ability of PrP^Sc ^to cross the intestinal tissues of rainbow trout when 139A was added to the mucosal side of everted or statically perfused intestine.

## Results and discussion

Our major aim was to investigate whether tissues of orally infected fish would retain some residual infectivity (i.e., not due to replication of TSE agents). Thus, turbot and trout were forced-fed with the 139A strain of scrapie and sacrificed from 1 to 90 days after challenge. Muscle, intestine, and brain were taken at different time points (1, 15, 30, 60 and 90 days post inoculation) and inoculated into recipient mice for the measurement of residual infectivity (Table 1A).

No behavioral or swimming abnormalities were observed in orally inoculated trout and turbot and histological examination of tissues from inoculated fish did not show any pathological mark nor prion protein was detected by immunohistochemical examination (data not shown). None of the recipient mice developed scrapie disease. No PrP^Sc ^was detected in the brain of the mice injected with turbot tissues (Table 1A). However, PrP^Sc ^was detected in the brain of one from the 8 mice inoculated with the intestine of trout taken one day after force-feeding inoculation, suggesting that the trout intestine contained some residual infectivity.

**Figure 1:**
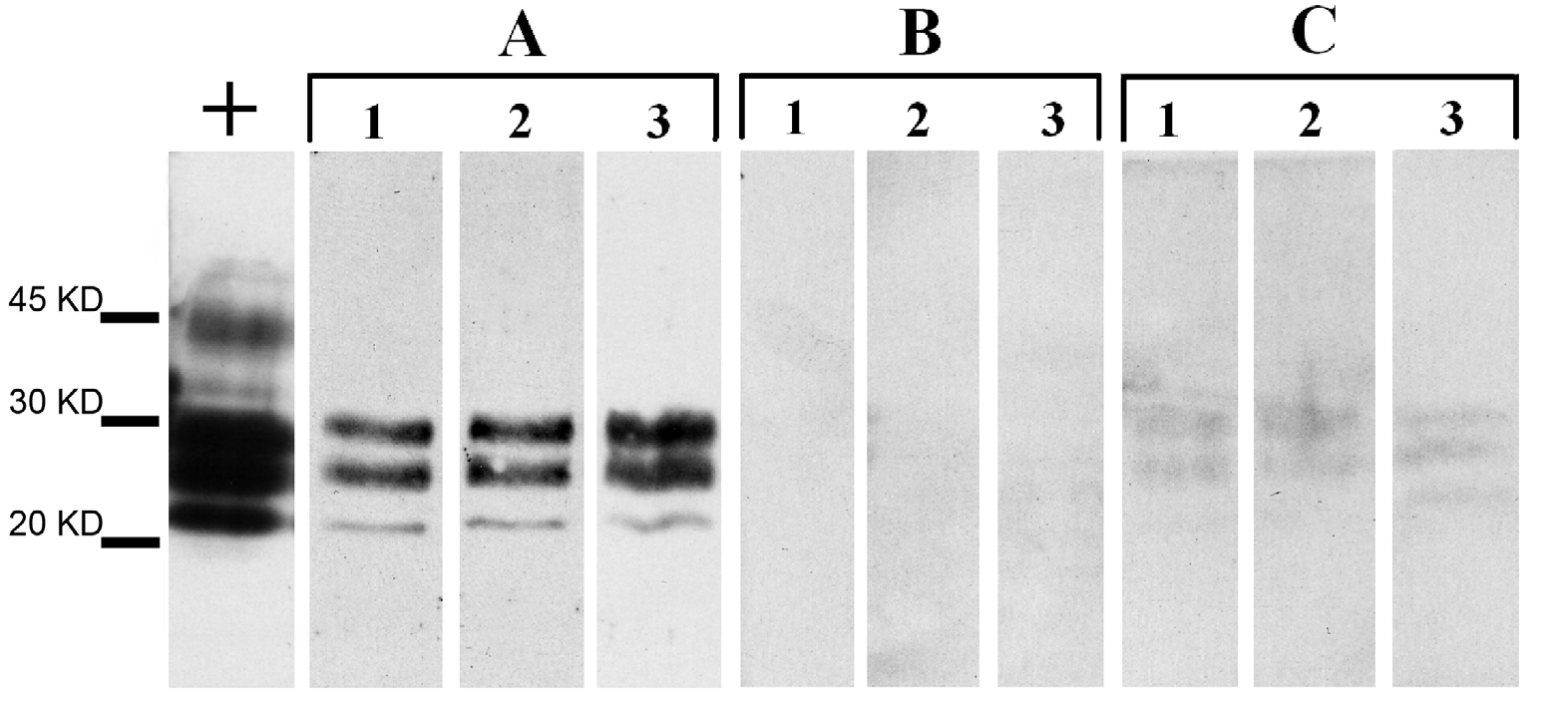
**PrP^Sc ^detection in the mucosal solution (A), the serosal solution (B), and the scraped mucosal layer (C) of trout everted intestine**. Western blot analysis of PrP^Sc ^in the crude mice brain homogenate (positive control, +), in the diluted mice brain homogenate solution (1:20) perfonding the mucosal side of the trout everted intestine (A), in the physiological solution fluxing the serosal side of the trout everted intestine (B), and in the scraped mucosal layer of the trout intestine (C). The mucosal tissue in sample C was obtained by gently washing (1 min) the intestine with a saline cold solution then ablated and homogenized 1 hour after perfusion (C). The experiment was repeated on three different trout intestine (1, 2, 3). The classical tri-banded pattern of diglycosylated, mono- and unglycosylated PrP^Sc ^was absent in B and only barely detectable in C suggesting a low absorption of PrP^Sc ^to the mucosal side of trout intestine and the absence of an active PrP^Sc ^transport through the intestinal barrier.

**Figure 2:**
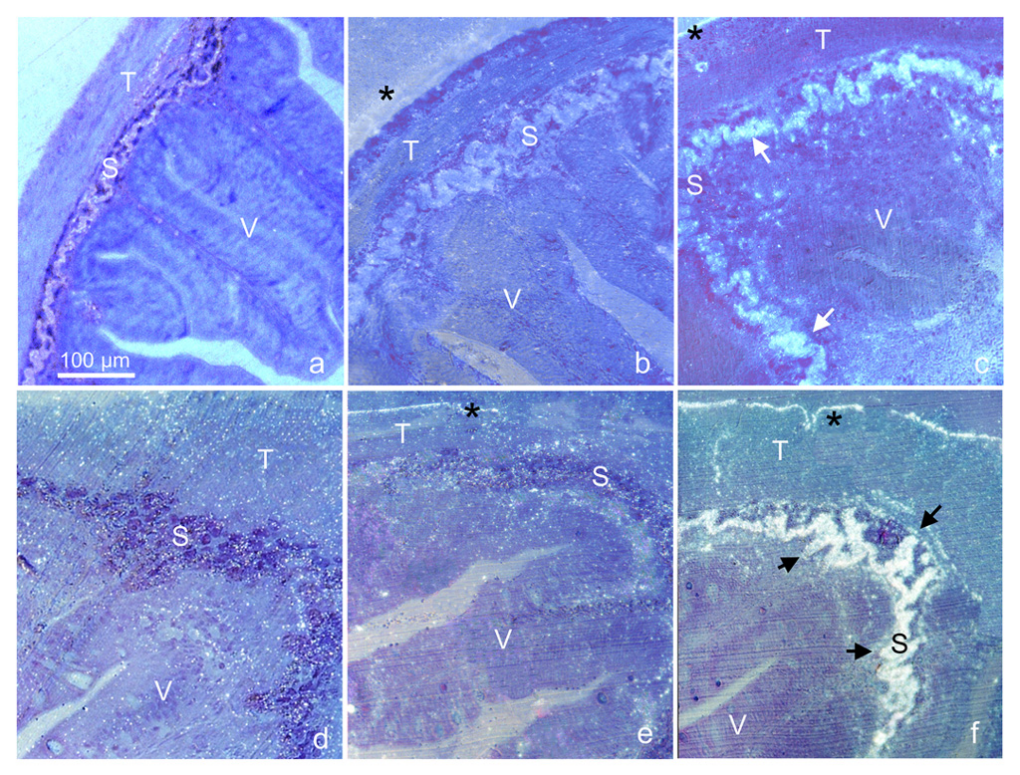
**PrP^Sc ^accumulation in the pyloric caeca (a, b, c) and non-everted intestine (d, e, f) of trout intestine statically perfused**. Immunohistochemistry on trout sections of pyloric caeca (a, b, c) and non-everted intestines (d, e, f). The static perfusion was performed for 1 hour at 15°C, in a PBS solution containing 50 μl/ml of either 10% uninfected mice brain homogenate (a, d) or 10% mice scrapie brain homogenate (139A) (b, c, e, f). Immunolabelling was performed with the monoclonal antibody SAF83 (a, c, d, f), or, as control, the monoclonal anti-HA against influenza virus (anti-HA, clone 12CA5, subtype IgG2b, k), (b, e). All the immunogold-labelled sections were silver enhanced and counterstained with 0.1% toluidine blue (see materials and methods for details). PrP^Sc ^localisation (arrows) occurred in the *stratum compactum *(S) of distal intestine and pyloric caecum incubated with SAF83 (c, f). No immunolabelling was present in control tissues incubated with the same antibody (a, d), except for a low unspecific background. The background was slightly higher, though unspecific, in anti-HA labelled sections (b, e) due to the high reactivity of this antibody. All micrographs were taken at the same magnifications; V = villo, T = *tunica muscolaris; *asterisks point to unspecific labelling present in all samples and due to micro-fractures between the outer specimen surface and the resin.

We therefore investigated whether trout intestine binds PrP^Sc ^and transfers it to the serosal side where it may spread to the lymphoid tissues and, eventually into the CNS. Using everted trout intestine immersed in a solution containing 139A we observed that PrP^Sc ^slightly absorbs to the mucosal intestinal layer as shown by the low, yet detectable signal at the western blot (Figure 1C). When statically perfused intestine was used in order to study the possible role of pyloric caeca in PrP^Sc ^absorption, it was possible to detect the presence of PrP^Sc ^by immunohistochemistry in the *stratum compactum *both in the trout intestine and in the pyloric caeca (Figure 2c, 2f). On the other hand, the absence of signal for PrP^Sc ^at the western blot in the solution fluxed into the serosal side of the everted intestine (Figure 1B) excludes, at least in this experimental setting, an active secretion of PrP^Sc ^from one side to the other side of the intestinal tract.

Such data are in agreement with the recent finding that stPrP is not detectable in the intestine of Rainbow trout [15], which renders unlikely an active uptake of exogenous PrP^Sc^, but does not exclude an unspecific binding of PrP^Sc ^to the fish intestinal mucosa.

In a second experiment, we inoculated trout and turbot with the same strain of scrapie as above by parenteral routes to increase the possibility of a successful infection (Table 1B). Brain and spleen, as the most likely tissues for PrP^Sc ^accumulation, were taken 15 and 90 days (the longest survival time of fish in a close circuit water tanks) after inoculation and residual infectivity was bio-assayed in mice. None of the recipient mice developed scrapie disease during their lifespan. However, PrP^Sc ^was observed at the western blot in the brain of a few recipient mice inoculated with tissues taken from turbot or trout (Table 1B). These data show that 15 days after inoculation only about one LD_50 _of scrapie-infectivity is present in the spleen of turbot, and much less in the brain of turbot or in the spleen of trout. Almost no infectivity (only 1 in 37 recipient mice were PrP^Sc ^positive) was however detected 90 days after inoculation of fish tissues, suggesting that the infectivity measured at 15 days post inoculation was likely related to the residual inoculum. As the fishes were not kept more than 90 days, it is not possible to exclude that infectivity might develop at a later time point, after an initial clearance phase of the inoculum, though this possibility is not likely.

**Figure 3:**
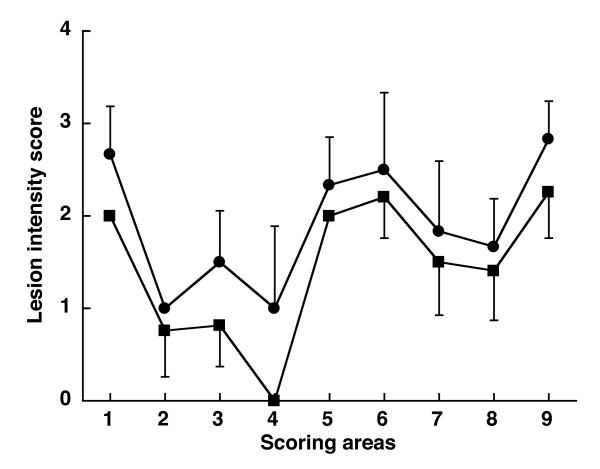
**Lesion profiles for mice following inoculation with 139A before (circle) and after (square) passage in fish**. The lesion profiles for the fish-passaged 139A (square) was a mean of 4 mice: 2 mice inoculated with turbot spleen at 15 days, 1 mouse inoculated with trout spleen at 15 days, and 1 turbot brain taken 90 days after parenteral inoculation. The reference curve (circle) was a mean of 6 mice inoculated with the 139A non passaged in fish. Vacuolation was evaluated in nine standard areas: 1, dorsal medulla; 2, cerebellar cortex; 3, superior colliculus; 4, hypothalamus; 5, thalamus; 6, hippocampus; 7, septum; 8, retrosplenial and adjacent motor cortex; 9, cingulate and adjacent motor cortex. Data are mean ± SE.

In our work, we did not evaluate whether mammalian TSE agents may establish infection in fish. Nonetheless, the "lesion profile" curves based upon the severity of spongiform changes in brain areas of mice injected with 139A before and after passage in fish (Figure 3), show a similar pattern suggesting that the 139A strain did not adapt to fish in the 3 months period examined. A statistical comparison of scores between the two curves, done area by area by the Mann-Whitney's test, showed a significant difference only in the dorsal medulla area (p = 0.045). No statistically significant difference was observed in the superior colliculus, hypothalamus, cingulate, and adjacent cortices. Permutation tests performed to overcome problems due to the small sample size, confirmed this pattern. The overall lower scores observed in the brain of mice inoculated with fish tissues could merely represent the fact that these mice were not symptomatic when sacrificed while control scrapie infected mice showed evident clinical signs of disease.

## Conclusion

These data show that about 4 million LD_50 _of 139A given by forced feeding were readily removed from fish intestine (both trout and turbot) in the first 24 hours after infection and that infection never reached the brain, the spleen or the muscles. This suggests that scrapie is quickly removed from fish tissues despite the presence of a cellular prion-like protein [15,18] and a prion protein-like gene in fish [11-13]. With all the cautions due to the difference between the 139A and the BSE strains, and that in this experiment fishes were observed for no more than 90 days after infection, it is tentatively possible to assume that the consumption of fish fed with BSE-infected MBM should not pose any substantial threat to public health.

## Methods

### "In vitro" experiments

Rainbow trout (*Oncorhynchus mykiss*) from 50 to 200 g of body weight were kept at 15°C for one week and starved 24 hours before the experiments. After sacrifice with a lethal dose of MS 222 (200 mg/l) the whole intestine was extracted, carefully everted, and canulated according to published procedures [19,20]. Briefly, canulated intestine was submerged in 25 ml of physiological solution (145 mM NaCl, 5 mM KCl, 2 mM MgSO_4_·7H_2_O, 7 mM CaCl_2_·2H_2_O, 10 mM NaHCO_3_, 5 mM D-Glucose, pH 7.2) containing scrapie mice brain homogenate (139A) to a final concentration of 5 mg/ml under constant agitation by air pump (sample A). The same solution, but without infected brain material (sample B), was fluxed through the canula inside the serosal side of the intestine by a peristaltic pump (1 ml/min) and collected for 1 h. After perfusion, the fish intestinal mucosa was abraded with a glass blade, homogenized in PBS (1:4 vol/vol), and further analysed for PrP^Sc ^detection by Western blot (sample C). Aliquots of 30 μl from samples A, B (sample B was lyophilised and resuspended in 1% wt/vol sarcosyl solution), and C were processed for PrP^Sc ^detection [21], using the mouse monoclonal antibody SAF83 (1:10000). This antibody was selected after screening different antibodies (SAF70, SAF32, 6H4, 8G8, SAF83, SAF84) for their ability to detect PrP^Sc ^on the fish mucosa homogenate. SAF70 and SAF84 showed cross reactivity with the fish mucosa, and 6H4 and 8G8 showed very low ability to detect PrP^Sc^. On the other hand, SAF32 and SAF83 were equally able to detect PrP^Sc ^with no cross reactivity with the fish mucosa.

Trout gastrointestinal tract, from oesophagus to anus, was submerged in sample A (see above) to be statically perfused. Samples were carefully washed and dissected in 10 mm segments. Cross sections, 1 mm thick, were randomly excised from each segment of the gastrointestinal tract, fixed in 0.8% glutharaldehyde, 3.0% formaldehyde in 0.01 phosphate buffer saline (PBS), dehydrated, embedded in hydroxyethyl-methacrylate (Kulzer Histo-Technik 8100, Germany) and serially cut. For immunostaining, sections were subjected to antigen retrieval (98% formic acid for 15 min or alternatively to microwave pretreatment at 600 W for 60 sec in PBS), before incubation with the primary antibody SAF83 at different dilutions. After incubation with a goat-anti mouse secondary antibody conjugated with 6 nm colloidal gold, the slides were rinsed and left to dry for one day before silver staining. Silver enhancement was performed with R-GENT kit for light microscopy (Aurion, The Netherlands). All samples were counterstained with 0.1% toluidine blue observed with epifluorescence (excitation 520–550 nm).

### "In vivo" experiment

Trout (*Oncorhynchus mykiss*) and turbots (*Scophthalmus maximus*) of 8 cm length were obtained from a commercial farm and acclimatized to laboratory conditions for 3 weeks before oral or parenteral infection with the mouse-adapted 139A strain of scrapie. The inoculum was prepared from a pool of 70 g of 139A scrapie-infected mouse (C57/BL) brains. Brain tissue was homogenized in sterile PBS to obtain a 10% (wt/vol) suspension, centrifuged at 913 × g (4°C per 15 minutes), and supernatant collected. Infectivity titre of this suspension was calculated by end-point titration as previously described [22], using groups of 6–10 animals for each dilution. The amount of infectivity, calculated by the Reed and Muench method, was 10^8.9 ^LD_50 _per gram of tissue. Control homogenate was prepared with the same procedure using brain tissue from healthy mice. One group of turbots (n = 20) or trout (n = 20) was force-fed with 0.05 ml of 139A scrapie-infected (10^6.6 ^LD_50_) or control mouse brain homogenates. Another group of turbots (n = 30) or trout (n = 30) was simultaneously inoculated by the intracerebral (0.03 ml, 10^6.4 ^LD_50_), intraperitoneal (0.1 ml, 10^6.9 ^LD_50_), and intramuscular (0.1 ml, 10^6.9 ^LD_50_) routes with either 139A or control brain homogenates. After inoculation, fish were maintained in close circuit water tanks under bio-security conditions to avoid water contamination with the scrapie agent.

Samples from brain, muscle, and intestine were taken from 3 orally-infected and 1 control fish for each species at 1, 15, 30, 60 and 90 days post-infection. Moreover, brain and spleen were taken from parenterally infected fish at 15 and 90 days post-infection. Collected samples were divided in two parts: one was immediately frozen at -80°C for measuring scrapie infectivity by mouse bioassay, the other was fixed for histological or immunohistochemical examination. Histopathological examination of fish tissues was routinely performed on every sample collected. Briefly, samples were fixed in Davidson and processed in an automatic tissue processor, embedded in paraffin and cut in 5 μm sections, deparaffinized, rehydratated and then stained with haematoxylin-eosin. The immunohistochemical detection of the prion protein in fish tissue sections was performed as described [23].

For the bioassay of fish tissues in mice, each group of fish samples, belonging to the same time point, was homogenized using separate sets of instruments to avoid accidental cross-contamination. Homogenisers were then accurately decontaminated (2 cycles of autoclave at 134°C for 1 hour followed by extensive washing and a third cycle of autoclave at 120°C for 20 minutes) before re-use on another group of samples. Twenty μl of 10% tissue homogenate were injected intracerebrally in groups of 6–11 C57/BL male mice for infectivity bioassay. Animals showing clinical signs (not necessarily related to scrapie) or moribund were sacrificed, their brains removed and divided in two halves. One half was immediately frozen at -70°C for PrP^Sc ^analysis, the other half was fixed in formalin for histological examination. PrP^Sc ^was purified as described [24] and detected by western blotting using monoclonal antibody SAF84 (SpiBio) diluted 1:5000. Mice were housed at the animal facility of the Istituto Superiore di Sanità (ISS) under the supervision of the Service for Biotechnology and Animal Welfare of the ISS who warrants the adherence to the national and international regulations on animal welfare.

### Histological analysis and lesion profiles of mice brains

Formalin-fixed brains were decontaminated with 98% formic acid for 1 h, stained with haematoxylin-eosin and scored for the intensity of vacuolar degeneration (ranging from 0, no vacuolar change, to 5, severe vacuolar change) in nine standard grey matter areas for building up the 'lesion profiles' [25]. Sections were coded and blind examined. In Figure 3, the lesion profile of 139A after passage in fish was a mean of 4 animals when brains for histology were available (2 mice inoculated with turbot spleen at 15 days, 1 mouse inoculated with trout spleen at 15 days, and 1 turbot brain taken 90 days after parenteral inoculation). We pooled these data together because none of these mice showed clinical signs and were sacrificed at about the same time after inoculation (221, 204, 215, and 221 days, respectively). The reference curve of 139A not passaged in fish was obtained pooling data from 6 mice. Immunohistochemistry was performed as described [23].

## Abbreviations

PrP: prion protein

PrP^c^: cellular prion protein

PrP^Sc^: pathological prion protein

stPrP: "similar to" PrP

TSE: Transmissible Spongiform Encephalopathy

CJD: Creutzfeldt-Jakob disease

GSS: Gerstmann-Sträussler-Scheinker disease

BSE: bovine Spongiform encephalopathy

MBM: bovine meat and bone meals

anti-HA: anti-haemagglutinin

## Authors' contributions

LI contributed to the design of the study, did mice inoculation, analysis of data and drafted the manuscript. BN, RA established the protocol for fish infection and sampling of fish tissues, they also performed it. AZDV, FF, and MI contributed to the "in vitro" experimental work and to the protocols setting. MS did the neuropathology of the mice brains and contributed to analysis of data. SB, ML, QL, and VV did western blot analysis of mouse brains and contributed to monitoring of mice, collection and analysis of data. FC did coordination and collection of clinical, biochemical and histological data from the mouse bioassay. SC contributed to the design of the study set up the protocol for the "in vitro" experiments. AF contributed to the design of the "in vivo " experimental work and the histological studies on fish tissues. MP participated in the design and coordination of the study, interpretation of data and drafting the manuscript. All the authors read and approved the final manuscript.

## Figures and Tables

**Table 1:** Measurement of scrapie infectivity in fish tissues by mouse bioassay. Brains from recipient mice inoculated with turbot or trout tissues from either oral or multiple parenteral routes were assayed for PrP^Sc ^by Western blot. Whenever samples were available, histology and/or immunohistochemistry was performed. Asterisks indicate mice contributing to the lesion profiles curve of the fish-passaged 139A (Figure 3)

A. Tissues from infected fish via the oral route								
	Turbot				Trout			
		
Days after infection	Muscle	Intestine	Brain	Spleen	Muscle	Intestine	Brain	Spleen

1	0/10	0/8	0/8	-	0/10	**1/8**	0/5	-
15	0/7	0/8	0/6	-	0/10	0/8	0/13	-
30	0/10	0/8	0/10	-	0/8	0/8	0/9	-
60	0/9	0/6	0/8	-	0/7	0/6	0/6	-
90	0/7	0/8	0/9	-	0/10	0/11	0/10	-

B. Tissues from infected fish via multiple parenteral routes								

15	-	-	**2/8**	4/7^*^	-	-	0/9	1/9^*^
90	-	-	1/7^*^	0/8	-	-	0/11	0/11
